# Prevalence of medical errors in Iran: a systematic review and meta-analysis

**DOI:** 10.1186/s12913-019-4464-8

**Published:** 2019-09-02

**Authors:** Siavash Vaziri, Farya Fakouri, Maryam Mirzaei, Mandana Afsharian, Mohsen Azizi, Morteza Arab-Zozani

**Affiliations:** 1 Department of Infectious Diseases, School of Medicine, Kermanshah University of Medical Sciences, Kermanshah, Iran; 2 Clinical Research Development Center, Imam Reza Hospital, Kermanshah University of Medical Sciences, Kermanshah, Iran; 3 School of Allied Medical Sciences, Kermanshah University of Medical Sciences, Kermanshah, Iran; 4 Department of Medical Microbiology, School of Medicine, Kermanshah University of Medical Sciences, Kermanshah, Iran; 5 Social Determinants of Health Research Center, Birjand University of Medical Sciences, Birjand, Iran

**Keywords:** Medical errors, Prevalence, Iran, Systematic review

## Abstract

**Background:**

Medical errors are considered as a major threat to patient safety. To clarify medical errors’ status in Iran, a review was conducted to estimate the accurate prevalence of medical errors.

**Methods:**

A comprehensive search was conducted in international databases (MEDLINE, Scopus and the Web of Science), national databases (SID, Magiran, and Barakat) and Google Scholar search engine. The search was performed without time limitation up to January 2017 using the MeSH terms of Medical “error(s)” and “Iran” in Endnote X5. Article in English and Persian which estimated the prevalence of medical errors in Iran were eligible to be included in this review. The JBI appraisal instrument was used to assess the quality of included studies, by two independent reviewers. The prevalence of medical errors was calculating using random effect model. Stata software was used for data analysis.

**Results:**

In 40 included studies, the most frequent occupational group observed were nursing staff and nursing students (21 studies; 52% of studies). The most reported type of error was medication error (25 studies; 62% of studies, with prevalence ranged from 10 to 80%). University or teaching hospitals (30 studies; 75% of studies) as well as, internal/intensive care wards (10 studies; 25% of studies) were the most frequent hospitals and wards detected. Based on the result of the random effect model, the overall estimated prevalence of medical errors was 50% (95% confidence interval: 0.426, 0.574).

**Conclusion:**

Result of the comprehensive literature review of the current studies, found a wide variation in the prevalence of medical errors based on the occupational group, type of error, and health care setting. In this regards, providing enough education to nurses, improvement of patient safety culture and quality of services and attention to special wards, especially in teaching hospitals are suggested.

**Electronic supplementary material:**

The online version of this article (10.1186/s12913-019-4464-8) contains supplementary material, which is available to authorized users.

## Background

Medical error(s) (MEs) are known as a inevitable event in the health system [1, 2]. MEs can occur in any care setting, including hospitals, health center, clinic, and laboratory, thus they can negatively effects on the patient safety [1–3]. There are various classification of MEs, technical errors, systematic errors (e.g., administrational organizational and process) and human errors (e.g., medication, diagnosis and treatment) [2]. According to various investigations at the global level, medication errors represents 10–18% of MEs, are placed in this category [1–5].

MEs increase the cost of hospitalization and medical expenses in both developed and developing countries which lead to decrease the quality of healthcare systems [1, 2].

Despite the development of diagnostic and treatment services, previous studies demonstrated an increase in ME events due to the complex issues of treatment and its timing [6–9]. According to different studies, the prevalence of MEs ranged from 1 to 40%. It has been estimated that about 17% of receptions in diagnostic and treatment centers lead to adverse events [7–12].

According to a review study in 2013, the prevalence of medication error in Middle Eastern countries including Iran is 7 to 90% [4].

Regarding this situation in Iran, some documents demonstrate the possibility of high MEs rate in the healthcare system of Iran. Based on the reports, one of 150 patients dies due to outcomes of MEs in hospitals [2, 9, 12].

Despite the high prevalence of this matter in health care settings, these errors are important challenges that threaten patient’s safety, but in almost half of the cases they were considered to be preventable with ordinary standards of care [13, 14].

Identifying the types of MEs and their prevalence has an important role in planning for prevention [2, 9]; so, having a detailed picture of this issue is very important, but given the lack of gold standards in this regard, the estimated prevalence varies from one study to another [2, 9, 10]. Therefore, the study of MEs in Iran is challenging and requires more attention.

To the best of our knowledge, there are no previous comprehensive studies with systematic methods on accessing the prevalence of MEs in Iran; therefore, due to the importance of ME events as a challenge in Iran’s health system, a need for comprehensive study are essential. Aim of the present study is to systematically review studies of the prevalence of MEs in Iran.

## Methods

The present study was done according to the Preferred Reporting Items for Systematic Reviews and Meta-analysis (PRISMA) statement [15].

### Search sources and search strategies

The literature search was conducted in international databases such as PubMed/MEDLINE, Scopus, EMBASE and Web of Science, national databases such as Scientific Information Database (SID) and Barakat and Google Scholar search engine using Persian and standard Medical Subject Heading (MeSH) English equivalent keywords without time limitation up to January, 2017. This process was done by the researcher that experts in databases searching. The search strategy used the following subject headings terms: “Medical error(s)” AND “Iran”. The search strategy used is listed in Additional file 1.

For each database, the search strategy was specified. Also, reference lists of all included studies were searched to identify any additional literature.

### Screening and selection criteria of articles

After comprehensive searching and removing the duplicate records in reference-management software (EndNote X5), remaining studies screened based on the title and abstract with consideration to inclusion and exclusion criteria.

Studies were accepted under the condition if they met following main inclusion criteria:

1. Purpose of the study states assessing the prevalence of MEs in Iran (in the present study, MEs defined as all recorded errors in different wards that were linked to treatment and caring processes of patients).
2. Articles published in the Persian and English languages.

The studies that weren’t part of primary research (such as articles related to conferences and letter to editor, case reports and review studies) or/and in unrelated field with the topic “prevalence of MEs” such as the cause and the risk factors of MEs, full text were not available and also non-English/Persian written studies were excluded from this review.

These exclusion criteria were also applied to the full-texts.

### Quality estimation and data extraction

The methodology quality of included studies was assessed by two independent reviewers using the standardized critical appraisal instrument for studies reporting prevalence data that prepared by the Joanna Briggs Institute (JBI).

The studies that obtained five or more “Yes” ratings out of nine were included in the review and studies with a minimum of five “yes” scores were excluded [16].

After quality appraisal, data from relevant studies were summarized in the designed extracted data form and entered the final extraction phase.

An Extracted data form was designed and piloted by the researcher and includes general information and characteristics of included articles such as title, author(s) and publication year and also the results of the study estimated the number of samples and main findings (prevalence of MEs).

Finally, relevant research on the prevalence of MEs in Iran was identified and the prevalence of MEs was extracted. The result reports and categorized based on three items:

A. According to involved individuals (physician, nurse, pharmacist, and patient).
B. According to the type of error.
C. According to the place of error (teaching hospitals or private centers).

### Assessment of publication Bias

Publication bias was investigated using a funnel plot. For funnel asymmetry the egger’s test was used to examine publication bias.

### Data analysis

I ^2^ statistics (with a significance level of I^2^ ≥ 50%) were used to assess between-studies statistical heterogeneity. Pooled estimates of cases with evidence of heterogeneity were performed by random effects model. Statistical analyses were conducted using STATA software, version 12 (Stata Corp. LP, College Station, TX, USA) and the level of significance in thisstudy is 5% (*p* < 0.05).

## Results

### Description of literature search

**Fig. 1:**
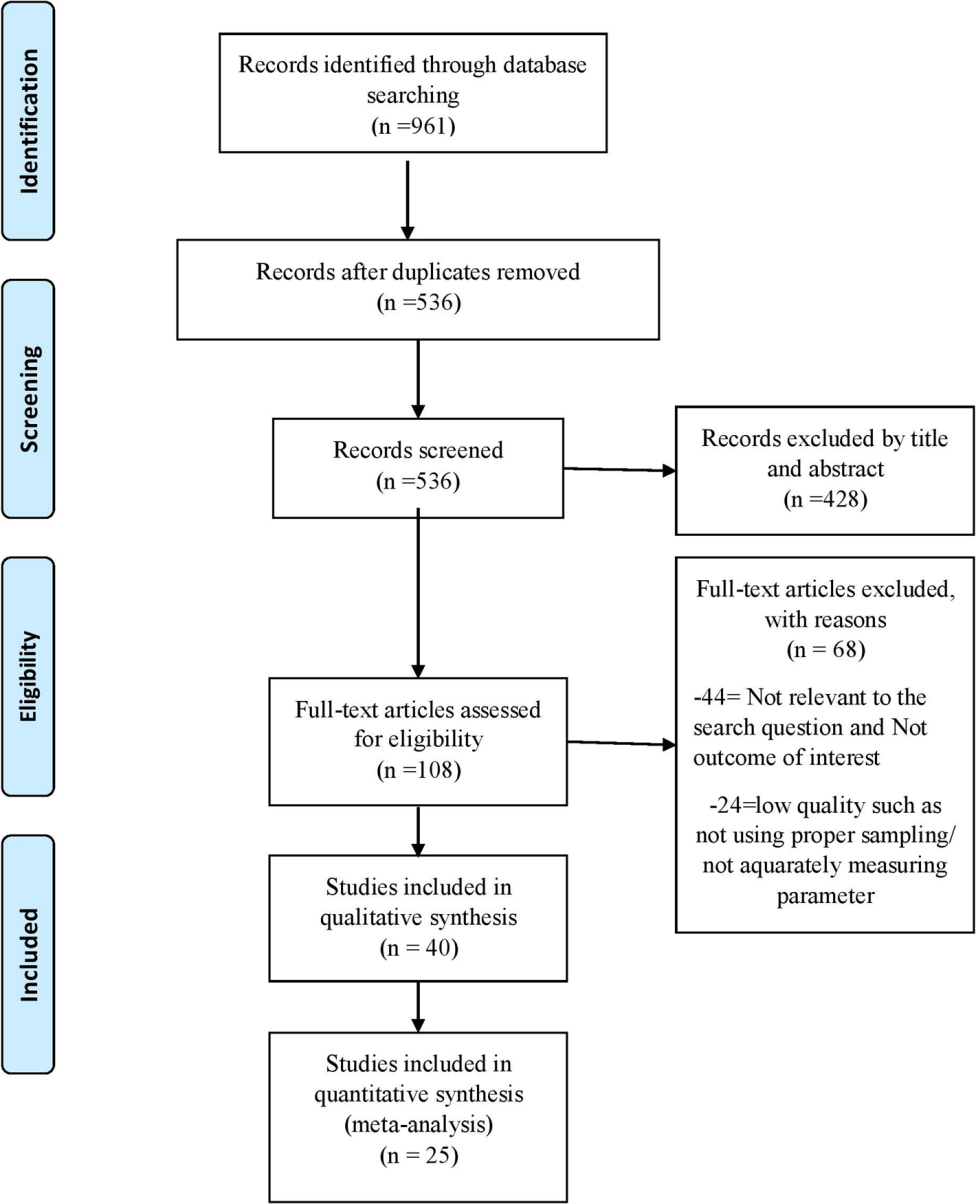
Flow diagram for study selection

Electronic searches of the database identified a total of 961 potentially relevant studies for study selection and, 425 duplicated were removed from the list. Out of 536 remained studies, 428 studies were excluded by screen of title and abstract. Based on assessment of the full text of 108 studies, 68 studies did not meet the inclusion criteria. After assessing the study quality, forty eligible studies were included in the review (Fig. 1).

### Description of quality estimation and the included studies

Forty studies scored between 5 to 8 out of 9, and were considered of an acceptable quality for inclusion in the qualitative synthesis (Additional file 2).

The summary characteristics of the included studies were shown in Additional file 3. Evidence showed all the studies reported at least one type of MEs. The prevalence of MEs in Iran varies widely and in some specific job groups, like nurses, are more than others. All studies were cross-sectional and performed in hospitals. In 40 studied, the lowest prevalence of MEs was reported by Gavgani et al. from Tabriz (16 medication errors) and the highest MEs was related to a study in Khammarnia et al. with 4379 errors [17, 18].

Four studies (10% of studies) reported the average prevalence of MEs [19–22]. From these studies, Yousefi et al., reported the highest average of medication error for each nurse in one-month duration from internal and surgical compartments (31.6 cases) and the lowest average of medication error reported in 3-month duration (1.33) by Hajibabaee and colleagues [19, 20].

### MEs according to involved individuals (physician, nurse, pharmacist and patients)

The prevalence of MEs was investigated in 14 and 26 articles, based on patients’ medical records and medical staff type such as nurses and physician, respectively. A total of 11,267 individuals participated in this study, of these, 542 were physicians and 3487 were nurses and 7238 were patients’ medical records [23–52]. The results showed that the prevalence of investigated MEs were much greater in nursing staff and nursing students than physicians. Thus, this staff was the most frequent populations under observation (21 studies; 52% of studies).

In this study, the highest prevalence of MEs is reported by physicians in research which performed in Kerman city on 293 physicians in 2015. In this study, the prevalence of MEs was 55% (270 cases) and the average age of participants in the study was 25.31 [53]. The lowest prevalence was reported by Sadr and colleagues in 2014 on 24 physicians [54]. The lowest prevalence of MEs among nurses was related to a study in Yazd city in 2015, in which the prevalence of MEs reported 47.9% (34 cases) [55]. Also, the highest prevalence of MEs among nurses reported in a study, which took place in one of the private hospitals in the northeast of Iran in 2014, and it reported the prevalence of ME in 572 cases [56].

### MEs according to the types of errors

In summary, studies covered a wide range of different types of MEs and some studies did not report the frequency of the type of MEs. Thirty three studies (82% of studies) reported the types of errors separately, in which 62% of the studies (25 of 40 studies) were only medication error. So, the commonly reported types of MEs were medication errors and prevalence of this type of MEs was between10 to 80%. In these studies, two dominant groups of medication errors were diagnosed, including prescribing/ordering and administration errors (prevalence ranged from 9.8 to 80.12% and 10 to 80%, respectively). The prevalence of error in drug consumption and prescription were greater for venous drugs. Among nurses, the most reported errors were medication prescription, in which the errors were related to injection phase and mistake in the method of injection and also giving wrong medication, wrong time and other cases.

In physician group, the main reported errors were related to functional, revision and errors associated with diagnosis, treatment, clinical examinations, error in prescription and record.

Other studies that related to medical records were included various ranges of MEs like errors acquired because of lack of expertise, technical and treatment errors, recording errors, and prescription errors. In addition, three studies used the Systematic Human Error Reduction and Prediction Approach (SHERPA technique) for detecting MEs. Overall estimation for MEs, as a human error identification technique, based on this technique were as follow; action (46 to 52%), checking (26 to 51%), communication (7 to 24%), retrieval (3 to 17%) and selection error (3 to 14%).

### MEs according to the place of error (university hospitals, private centers)

The most common investigated place of error was related to academic/university/teaching hospitals (30 studies; 75% of studies). The other studies were related to private hospitals, social security and military centers (10 studies; 25% of studies). Only 20 studies (50% of studies) were presented necessary information for prevalence of MEs occurrence in a separate manner for hospital wards and the place of error occurrence. From this, 10 studies performed in internal and ICU ward and the rest of studies (10 studies) were conducted in the emergency room and surgical wards. It was also variable in different wards of observation (Additional file 1).

In some studies, the occurrence of MEs compared between various wards of hospitals. In which, the prevalence of error was reported higher in internal, ICU and surgical wards.

### The results of the meta-analysis

After a comprehensive search and quality assessments of included studies, forty studies were included in the qualitative synthesis, of which twenty-five were considered for quantitative synthesis; fifteen studies had insufficient data for quantitative analysis, and their results are summarized qualitatively.

**Fig. 2:**
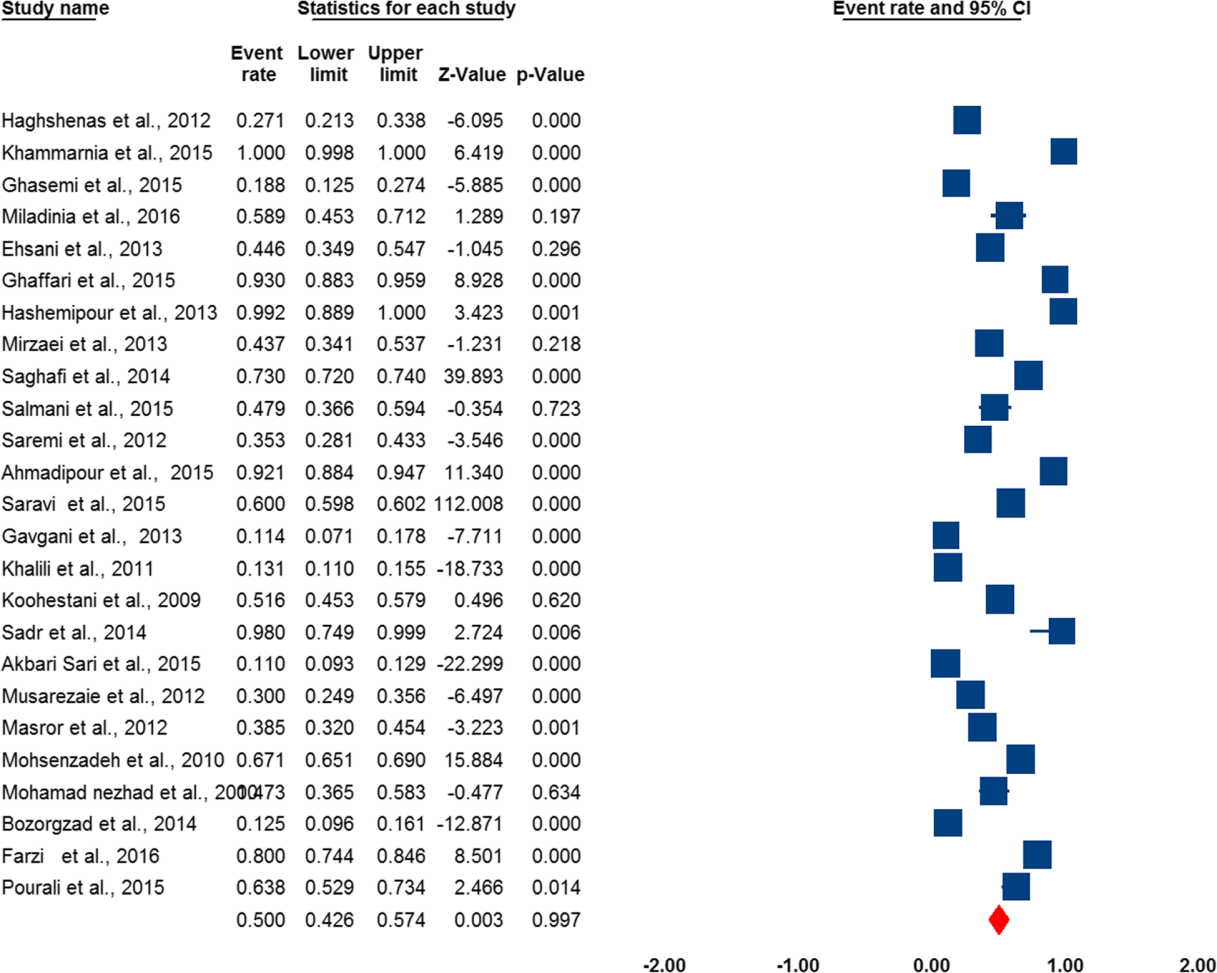
Forest plot of the random-effect meta-analysis for prevalence of medical error (s) in Iran

The results of I^2^ were showed the heterogeneity of the studies (I^2^ = 99.1%, *p* < 0.001). The prevalence of MEs was 50% (95% CI: 0.426 to 0.574). The results of the random-effect meta-analysis for prevalence of MEs in Iran have been represented in Fig. 2.

**Fig. 3:**
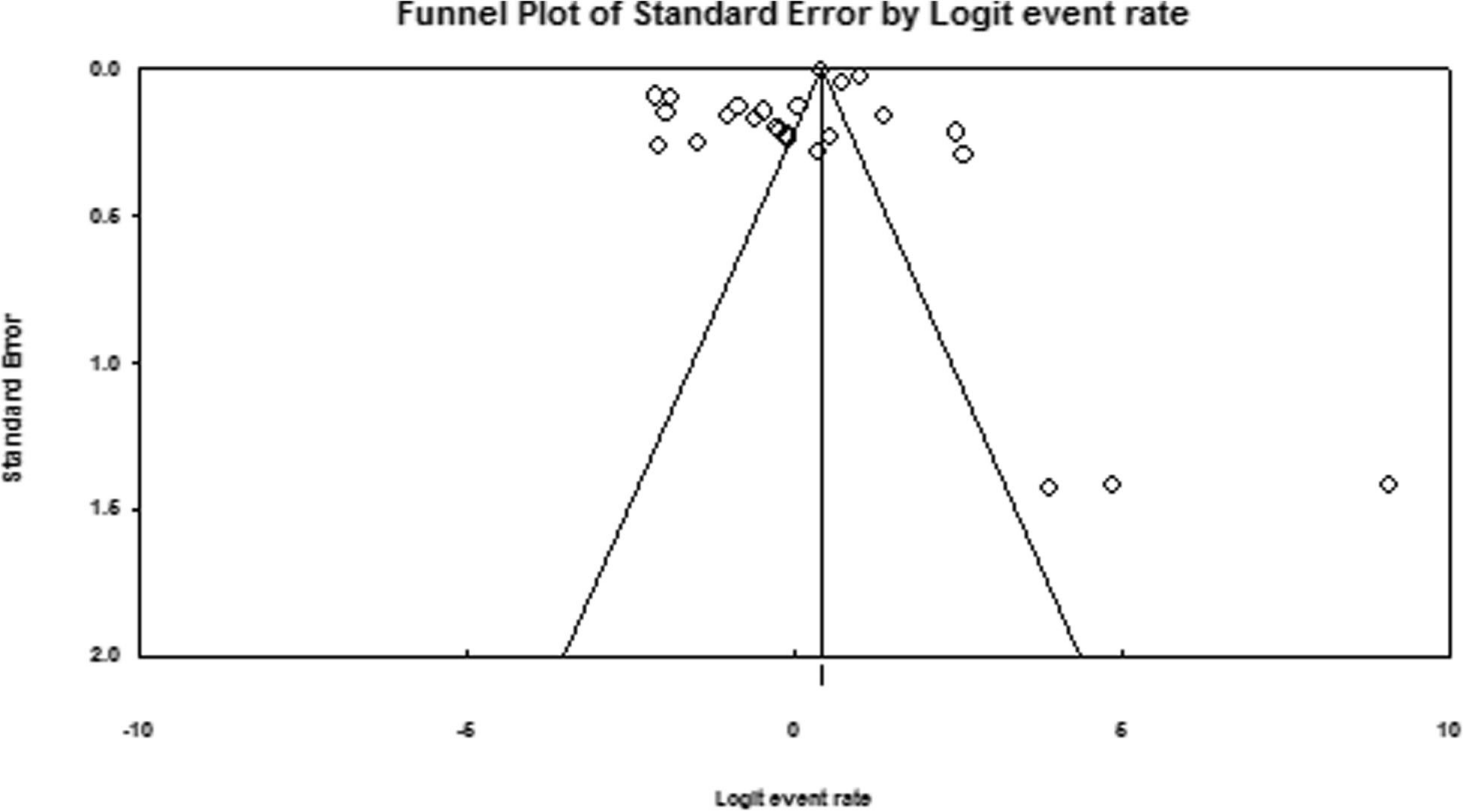
Funnel plot to assess publication bias for the prevalence of medical error (s) in Iran

Additionally, the funnel plot of overall MEs did not show any evidence of publication bias; (*P* value of Egger’s test = 0.236) (Fig. 3).

**Fig. 4:**
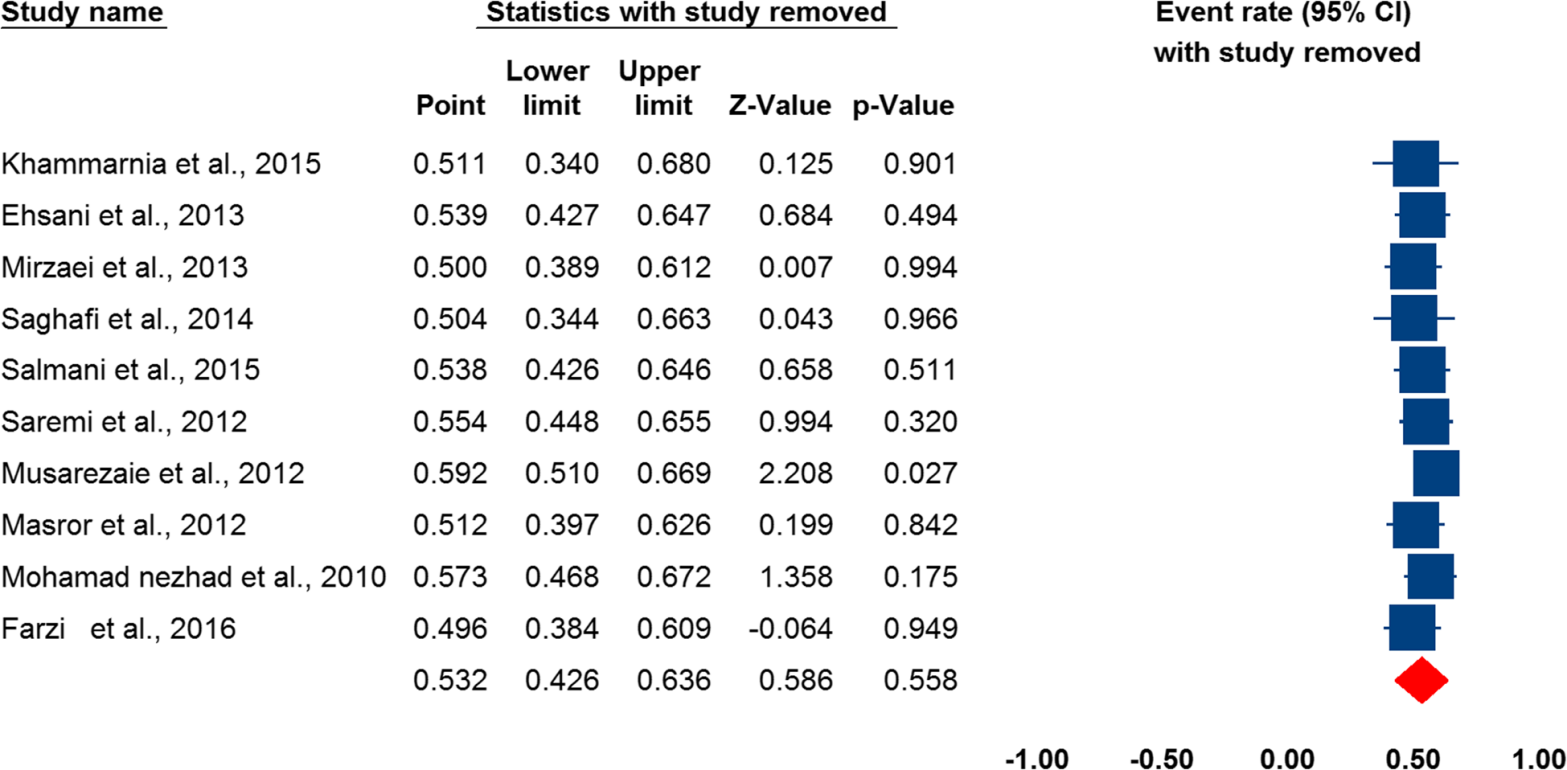
Forest plot of the prevalence of medical errors in Iran, by nurses

**Fig. 5:**
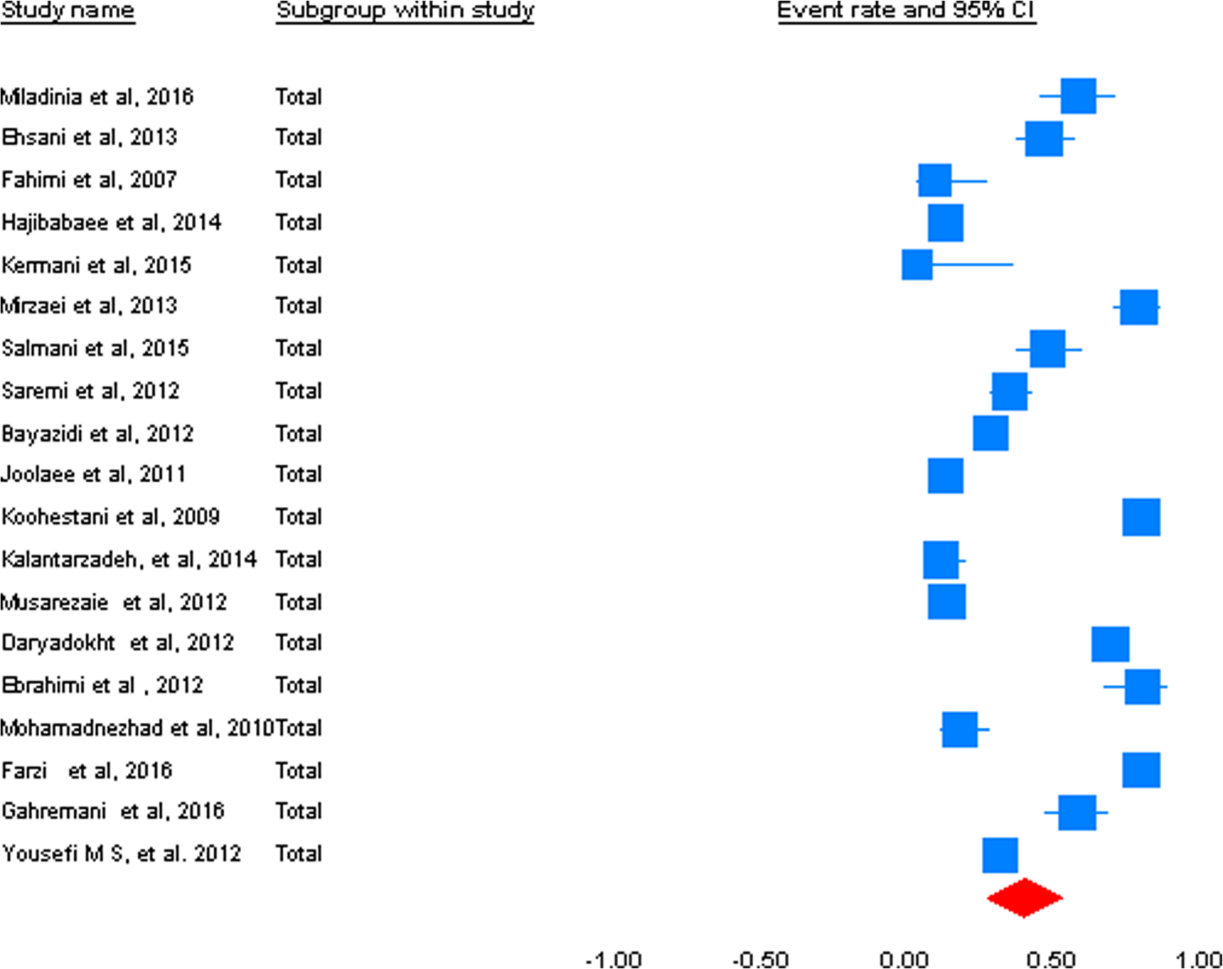
Forest plot of the prevalence of medication errors in Iran

**Fig. 6:**
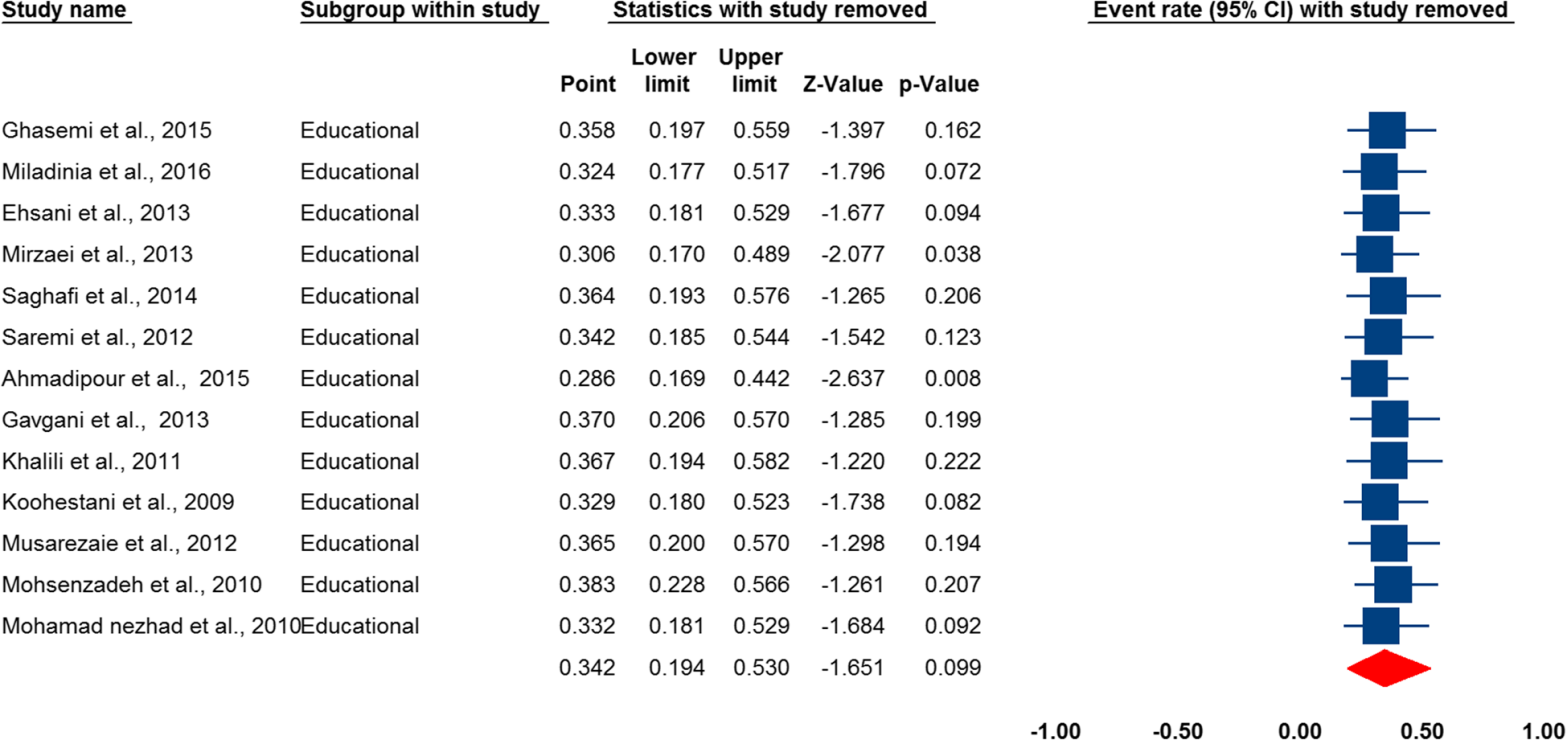
Forest plot of the prevalence of medical errors in educational hospitals

Additionally, for investigating the potential sources of heterogeneity we recheck the data and it seems that the heterogeneity is due to clinical diversity like variation in occupational group, type of error, and health care setting, then, we conduct a subgroup analysis based on participants, settings, and type of errors (Figs. 4, 5 and 6).

According to the results of subgroup analysis, the highest prevalence of MEs in Iran observed in nursing staff (pooled estimate = 0.532, 95% CI: 0.426, 0.636), and teaching hospitals (pooled estimate = 0.342, 95% CI: 0.194, 0.530). Medication error was the most common type of MEs (pooled estimate = 0.392, 95% CI: 0.271, 0.528). In subgroup analysis, heterogeneity not found in studied included (data not shown).

## Discussion

### Prevalence of MEs

Based on the systematic review of the current studies, the prevalence of MEs in different researches of Iran had a wide variation. Results of the meta-analysis, indicate that the overall prevalence of ME(s) was 50% (95% confidence interval: 0.426, 0.574).

The results of studies included in this systematic review on prevalence of MEs were divided and investigated in three general categories, including the health specialty/profession, the place of error occurrence/healthcare setting, and the type of the error.

### Differences in the prevalence of MEs among health professionals (providers)

The range of reported prevalence of MEs from all included studies in this review was wide. The prevalence of MEs was highest among nurses compared with the other health professionals (53%, with a range of 43–66%), but it should be noted that the methods of collecting data were self-reporting questionnaires. Some obstacles such as fear of the lawsuit in clinical and health staff, social desirability bias can lead to the lower/upper prevalence of occurrence and number of errors in these studies and lead to under or over reporting [57, 58].

This issue consist with the findings reported by Mansouri et al. [9]. The reason for the higher prevalence of errors among nurses can obtain the higher exposure to job damage due to heavy job load, lack of night sleep and high stress that can make the occurrence of errors and decrease the treatment and care quality in this job group [58, 59]. Nurses are the largest community of health care professional. Thus, organizational factors such as organizational culture, organizational structure and organizational policies can be other influential factors in the issue of reporting rate of nurses’ error [2, 59]. Furthermore, a review study by Koller in 2016 showed nurses had more tendencies toward reporting the occurrence of errors in respect to the physician [60].

### Differences in the prevalence of MEs based on type of error

The most common reported type of MEs was medication errors in selected articles (39%, with a range of 27–53%). According to these studies, the main type of reported medication errors, summarized in three types of errors, including prescription error like a wrong dosage, and injection, prescribing wrong drug and prescribing a drug without considering the proper time (oral or injection), administration error and transcription errors. In these studies, the prevalence of errors in medication prescription reported to be much higher in injective medication than other drugs, and our findings are in the accordance with previous studies [4, 61, 62].

In a review study conducted by Mansouri and colleagues, personnel of nursing and students of nursing were the dominant groups in the occurrence of medication errors, and the main medication errors were because of insufficient knowledge about medication [9].

Different studies in various countries investigated the MEs including medication errors, and their findings are in acceptance with the present study. In different studies, prescription errors like wrong dosage and lack of knowledge about drugs were always the main types of medication errors and most of them investigated the medication errors during prescription phase in Iran [3, 8, 9, 62–66].

In recent years, many studies were associated with medication errors. The results of the present study are very close to similar studies in this field. According to the reports, medication errors are the most common type of MEs [4, 65–67]. For example, Karthikeyan and colleagues in a study with the title, ‘systematic review of medication errors’ reported that the rate of occurrence of errors in drug prescription phase is 7.1 to 68.2%, which is in accordance with present study [62].

A systematic review conducted by Mansouri and his colleagues, which, reviewed systematic medication errors in Iran. Although they analyzed the source of errors, preventing strategies, the reason for under-reporting of error, prevent medication errors and common drugs in medication errors. They also reported a similar result compared to our results [9, 66]. Results of a review done by Mansouri and colleagues indicated the prevalence of medication errors at different stages was as follows: prescribing (47.8%), transcribing (51.8%), dispensing (33.6%) and administration (70%) [9].

In another systematic review study, 45 studies in the field of medication error included, among them 13 studies were from Iran. In this study, the mistakes in prescription medication were the most common MEs, and the most common errors of prescribed medication were the wrong dosage with the occurrence of 0.34% [4].

According to the study by Matin et al., the overall estimated 1-year period prevalence of medication errors and its reporting rate to nurse managers among nurses were 53% (95% confidence interval, 41–60%) and 36% (95% confidence interval, 23–50%), respectively [67].

From another similar field of study, we could refer to a systematic review study by Santesteban and colleagues in 2015, who reported the highest medication errors, were belonged to transcription, administration and prescribing errors. Also, mistakes in given dosages in prescribed drugs were the dominant medication errors [64] and our findings are close to these results. From the findings of this study and previous research in this field, we can conclude Iran has a high prevalence of medication errors. Hence, in the case of minimizing prescribing drug errors, we need to use risk management methods.

### Differences in the prevalence of MEs based on healthcare setting

The findings show that academic/university/teaching hospitals had higher level of reporting of occurrence of MEs (34%, with a range of 19–53%). Also, internal wards, ICU, and surgical wards had higher level of reporting of MEs. However, the data for the third aim was only accessible for 30% of studies. Shortage of studies in this field may mislead the true error occurrence in association with departments. A systematic review study by Mansouri reported similar results in associated with the present study. This study reported that medication error was investigated frequently in ICU, internal ward and surgery ward [9, 66]. In general, in Iran’s hospitals, the highest prevalence of MEs occurred in general and larger hospitals [2].

A meta-analysis that conducted in ICU unit in 2015 showed that ICU wards are prone environments for the occurrence of MEs [1]. This difference in error occurrence in various wards of the hospitals because of matters such as the certain nature of patients, diagnostic devices, and hard job [68, 69].

Based on the literature, there was no studies that review types of MEs and most of the studies obtain a systematic review in medication errors’ field. So, performing studies with use of specific definition in MEs is suggested to gain reliable and valuable results for future programming.

### Limitations of our overview

This quantitative synthesis is subject to some limitations. The pooled estimate of the prevalence of MEs, calculated based on twenty-five of the reviewed studies. Thus, for all the included studied, a meta-analysis could not be performed due to inadequate or incomplete data. Missing data and insufficient evidence made it difficult to provide an accurate estimation of the true prevalence.

We can refer to low quality and inability to look for combinatorial keywords in internal database sources as other limitations of the present study, in which all the studies didn’t use a united method or standard measurements for their variables. Also, because of the low quality research, their data didn’t enter the present study. Some of the articles excluded from this study because they didn’t present a true and clear explanation of MEs. Variable methodological quality, mostly related to the outcome measures used, should be considered.

Other limitations included the heterogeneity and variability between studies, mixed denominators (some studies report the prevalence of MEs based on the number of participants, while others based on opportunity of errors). The current study was conducted over a limit period of time which may not have been representative of all MEs events. It is likely that studies further to 2017 may have contained useful evidence, because of we did not make attempts to updating the literature search strategy; therefore likely to have missed all of the more recent literature published should be considered and the results need to be interpreted cautiously.

## Conclusion

Result of the comprehensive literature search of the current studies, found a wide variation in the prevalence of medical errors based on the occupational groups, type of error, and healthcare setting. In this regards, providing enough education to nurses, improvement of patient safety culture and quality of services and attention to special wards, especially in teaching hospitals are suggested.

Additionally, more researches are needed to clarify the nature of MEs, effective factors in the prevalence of MEs and the outcome of MEs.

## Additional files

Additional file 1: Search strategy in databases. (DOCX 13 kb)

Additional file 2: Critical appraisal results of the included studies. (DOCX 42 kb)

Additional file 3: Detailed summary of the outcome measures and results of included studies. (DOCX 43 kb)

## Data Availability

The datasets supporting the conclusions of this article are included within the article and its Additional files.

## Abbreviations

MEs: Medical error(s)
MeSH: Medical Subject Heading
PRISMA: Preferred Reporting Items for Systematic Reviews and Meta-analysis
SHERPA technique: Systematic Human Error Reduction and Prediction Approach
STROBE: Strengthening the Reporting of Observational Studies in Epidemiology
