# Supplementary material for: Prevalence of medical errors in Iran: a systematic review and meta-analysis
Source: BMC Health Serv Res. 2019 Sep 2;19:622. doi: 10.1186/s12913-019-4464-8 (PMC6720396; doi:10.1186/s12913-019-4464-8)
Supplement: Supplementary file 1 — Search strategy in databases. (DOCX 13 kb) [file 12913_2019_4464_MOESM1_ESM.docx]

**Additional file 1: Search strategy in databases**

|  | Search Terms | Search Statement | Results |
| --- | --- | --- | --- |
| Using international database | #1 ‘‘Iran’’ [Title/Abstract]  #2 ‘‘Medical error’’ [MeSH] [Title/Abstract] | #3 #1 AND #2 | Using PubMed Database=70  Using Embase Database =63  Using Scopus Database=322  Using Web of Science Database=11  Using Google Scholar search engine =67 |
| Using national database | Persian standard keywords |  | Using Scientific Information Database (SID)=40  Using Barakat Database=388 |
