# Supplementary material for: Prevalence of medical errors in Iran: a systematic review and meta-analysis
Source: BMC Health Serv Res. 2019 Sep 2;19:622. doi: 10.1186/s12913-019-4464-8 (PMC6720396; doi:10.1186/s12913-019-4464-8)
Supplement: Supplementary file 2 — Critical appraisal results of the included studies. (DOCX 42 kb) [file 12913_2019_4464_MOESM2_ESM.docx]

**Additional file 2: Critical appraisal results of the included studies**

| Citation | Q1 | Q2 | Q3 | Q4 | Q5 | Q6 | Q7 | Q8 | Q9 | Total score |
| --- | --- | --- | --- | --- | --- | --- | --- | --- | --- | --- |
| Haghshenas et al., 2012 [[52](#_ENREF_52)] | Y | U | Y | Y | Y | Y | N | Y | Y | 7 |
| Khammarnia et al., 2015 [[18](#_ENREF_18)] | Y | Y | Y | Y | Y | Y | U | Y | Y | 8 |
| Ghasemi et al., 2015 [[51](#_ENREF_51)] | Y | U | Y | Y | Y | Y | U | U | Y | 6 |
| Miladinia et al., 2016 [[50](#_ENREF_50)] | Y | Y | Y | Y | Y | Y | U | Y | Y | 8 |
| Ehsani et al., 2013 [[49](#_ENREF_49)] | Y | Y | Y | Y | Y | Y | U | Y | Y | 8 |
| Fahimi et al., 2007 [[48](#_ENREF_48)] | U | Y | U | Y | U | N | Y | Y | Y | 5 |
| Fahimi et al., 2009 [[47](#_ENREF_47)] | Y | N | N | Y | Y | N | Y | Y | Y | 6 |
| Ghaffari et al., 2015 [[46](#_ENREF_46)] | Y | Y | Y | Y | Y | U | U | Y | Y | 7 |
| Hajibabaee et al., 2014 [[19](#_ENREF_19)] | Y | Y | Y | Y | Y | Y | U | Y | Y | 8 |
| Hashemipour et al., 2013 [[45](#_ENREF_45)] | Y | Y | Y | Y | U | N | Y | Y | U | 6 |
| Kermani et al., 2015 [[44](#_ENREF_44)] | Y | Y | Y | Y | U | Y | Y | U | U | 6 |
| Mirzaei et al., 2013 [[43](#_ENREF_43)] | Y | Y | Y | Y | Y | Y | U | Y | Y | 8 |
| Mousavi et al., 2012 [[42](#_ENREF_42)] | Y | U | U | Y | Y | U | Y | Y | Y | 6 |
| Saghafi et al., 2014 [[41](#_ENREF_41)] | Y | U | Y | U | U | Y | Y | Y | Y | 6 |
| Salmani et al., 2015 [[55](#_ENREF_55)] | Y | Y | Y | U | U | Y | Y | Y | U | 6 |
| Saremi et al., 2012 [[40](#_ENREF_40)] | Y | N | Y | N | Y | N | N | Y | Y | 5 |
| Vazin et al., 2012 [[39](#_ENREF_39)] | Y | N | N | N | Y | Y | U | Y | Y | 5 |
| Ahmadipour et al., 2015 [[53](#_ENREF_53)] | Y | Y | Y | U | Y | Y | Y | Y | Y | 8 |
| Bayazidi et al., 2012 [[21](#_ENREF_21)] | Y | Y | Y | Y | Y | Y | U | Y | Y | 8 |
| [Saravi](https://www.ncbi.nlm.nih.gov/pubmed/?term=Saravi%20BM%5BAuthor%5D&cauthor=true&cauthor_uid=25870528)  et al., 2015 [[38](#_ENREF_38)] | Y | N | Y | Y | Y | Y | U | Y | Y | 7 |
| Gavgani et al., 2013 [[17](#_ENREF_17)] | Y | Y | Y | N | Y | Y | N | U | Y | 6 |
| Joolaee et al., 2011 [[22](#_ENREF_22)] | Y | Y | Y | Y | Y | Y | U | Y | Y | 8 |
| Khalili et al., 2011[[37](#_ENREF_37)] | Y | Y | U | Y | U | Y | U | Y | Y | 6 |
| Koohestani et al., 2009 [[36](#_ENREF_36)] | Y | Y | Y | Y | U | Y | U | Y | Y | 7 |
| Sadr et al., 2014 [[54](#_ENREF_54)] | Y | Y | U | Y | Y | N | U | Y | Y | 6 |
| Akbari Sari et al., 2015 [[35](#_ENREF_35)] | Y | Y | U | Y | U | Y | Y | N | U | 5 |
| Mohammadfam et al., 2015 [[33](#_ENREF_33)] | Y | Y | U | N | U | Y | N | Y | Y | 5 |
| Kalantarzadeh et al., 2014 [[34](#_ENREF_34)] | Y | N | Y | Y | N | Y | N | Y | Y | 6 |
| Valizadeh et al., 2008 [[32](#_ENREF_32)] | Y | Y | U | Y | N | Y | U | Y | Y | 6 |
| Musarezaie et al., 2012 [[31](#_ENREF_31)] | U | Y | Y | Y | U | Y | N | Y | Y | 6 |
| Masror et al., 2012 [[30](#_ENREF_30)] | Y | Y | Y | Y | U | Y | N | Y | Y | 7 |
| Mohsenzadeh et al., 2010 [[29](#_ENREF_29)] | U | Y | Y | Y | U | Y | N | Y | Y | 6 |
| Ebrahimi et al., 2012 [[28](#_ENREF_28)] | Y | N | N | N | U | Y | Y | Y | Y | 5 |
| Tabatabae et al., 2014 [[56](#_ENREF_56)] | U | Y | Y | Y | U | Y | Y | Y | Y | 7 |
| Mohamad Nejad et al., 2010 [[27](#_ENREF_27)] | U | Y | Y | N | U | Y | Y | Y | Y | 6 |
| Bozorgzad et al., 2014 [[26](#_ENREF_26)] | Y | Y | Y | N | N | Y | N | Y | Y | 6 |
| Farzi et al., 2016 [[25](#_ENREF_25)] | Y | Y | Y | Y | N | Y | N | Y | Y | 7 |
| Pourali et al., 2015 [[24](#_ENREF_24)] | U | Y | U | Y | N | Y | Y | Y | Y | 6 |
| Gahremani et al., 2016 [[23](#_ENREF_23)] | Y | Y | Y | N | Y | Y | Y | U | Y | 8 |
| Yousefi et al., 2012 [[20](#_ENREF_20)] | Y | Y | Y | N | N | Y | Y | Y | Y | 7 |
| % | 34 | 30 | 29 | 28 | 19 | 33 | 15 | 35 | 36 |  |

N, no; U, unclear; Y, yes.
Q1: Was the sample frame appropriate to address the target population?
Q2: Were study participants sampled in an appropriate way?
Q3: Was the sample size adequate?
Q4: Were the study subjects and the setting described in detail?
Q5: Was the data analysis conducted with sufficient coverage of the identified sample?
Q6: Were valid methods used for the identification of the condition?
Q7: Was the condition measured in a standard, reliable way for all participants?
Q8: Was there appropriate statistical analysis?
Q9: Was the response rate adequate, and if not, was the low response rate managed appropriately?
