# Supplementary material for: Prevalence of medical errors in Iran: a systematic review and meta-analysis
Source: BMC Health Serv Res. 2019 Sep 2;19:622. doi: 10.1186/s12913-019-4464-8 (PMC6720396; doi:10.1186/s12913-019-4464-8)
Supplement: Supplementary file 3 — Detailed summary of the outcome measures and results of included studies. (DOCX 43 kb) [file 12913_2019_4464_MOESM3_ESM.docx]

N**Additional file 3: Detailed summary of the outcome measures and results of included studies**

| **Study /date** | **Sample** | **Prevalence of medical error** | **Type of medical error** | | **Study location** | **study settings** | |  |
| --- | --- | --- | --- | --- | --- | --- | --- | --- |
| Haghshenas et al.,  2012 [[52](#_ENREF_52)] | N=192 record | 52 (27.1%) | ----- | | ----- | forensic medicine | |  |
| Khammarnia et al., 2015 [[18](#_ENREF_18)] | N= 4379 | 4379 | Systemic errors 1189(27.1%);  Treatment=771 (17.6);  drug=754 (17.2);  record=526 (12.0); Technical=899 (20.5) | | ----- | Internal wards/  Operation room)/surgery | |  |
| Ghasemi et al., 2015 [[51](#_ENREF_51)] | N=106 (39 Physician  69 nurse) | 199 errors | Action=93 (46.8%); checking=51 (25.6%); communication=24 (12.1%); retrieval= 17 (8.5%); selection=14 (7%) | | Educational hospital | acute care unit | |  |
| Miladinia et al., 2016 [[50](#_ENREF_50)] | N=53 Pediatric Nurses | 131 Medication error | Wrong dose (36.6%); wrong drug preparation (14.5%);  Wrong infusion velocity (11.45%) | | Educational hospital | pediatric ward | |  |
| Ehsani et al., 2013 [[49](#_ENREF_49)] | N=94 nurses | 42 (46.8%) | Infusion rates=14 (33.3%)  Administering two doses of medicine instead of one=10 (23.8%)  Omission of medicine=6(14.2%) | | Educational hospital | emergency department | |  |
| Fahimi et al., 2007 [[48](#_ENREF_48)] | N=28 nurses | 380 (9.4%)  medication error | Fast bolus administration=189(49.7%)  Wrong infusion rate=100(26.3%)  Wrong dose or diluent calculation=38(10) | | Educational hospital | ICU | |  |
| Fahimi et al., 2009 [[47](#_ENREF_47)] | N=287 medical chart | 167 (29.9%) medication error | omitted doses 149(52%)/ Wrong dose 53(18)/  Requesting drug more than required 20(7%)/  Alternative drug 20(7%)/  Unauthorized medication() | | Educational hospital | ----- | |  |
| **Study /date** | **Sample** | **Prevalence of medical error** | **Type of medical error** | **Study location** | | | **study settings** |  |
| Ghaffari et al., 2015 [[46](#_ENREF_46)] | N= 183 reception staff | 1188 errors | Incorrect patient information entry=174 (14.6%)  Error accepting requested items in patient's checklist=147(12.4%)  Inappropriate sampling rate=120(10.1%)  Error entering results=78(6.6%)  Other errors= 519(43%) | Educational hospital /Private  hospitals | | | clinical laboratories |  |
| Hajibabaee et al., 2014 [[19](#_ENREF_19)] | N= 300 nurses | mean number of reported=1.3 | Administration error | teaching and non-teaching hospitals | | | medical, surgery, orthopaedic, gynaecology obstetric wards |  |
| Hashemipour et al., 2013 [[45](#_ENREF_45)] | N=64 case | 1127 medical malpractices | ------ | Medical Council and Legal Medicine Organization | | | dentists |  |
| Kermani et al., 2015 [[44](#_ENREF_44)] | N=15 (nurses and physicians) | 359 errors including | Action (52.65%)/  Selection (8.9%)  Checking (45.2)  retrieval (7.11%) communication (3.5%) | Educational hospital /Private  hospitals | | | ----- |  |
| Mirzaei et al., 2013 [[43](#_ENREF_43)] | N= 96 Nurses | 79.2% | -giving oral drugs by mistake=51(53.1%)  -medication later or earlier than the stipulated time=40(41.7%) | an educational hospital | | | ----- |  |
| Mousavi et al., 2012 [[42](#_ENREF_42)] | N= 450 patients | 596 IV fluid therapy errors | ----- | Educational hospital | | | infectious diseases wards |  |
| **Study /date** | **Sample** | **Prevalence of medical error** | **Type of medical error** | **Study location** | | | **study settings** | |
| Saghafi et al., 2014 [[41](#_ENREF_41)] | N= 565 patients | 15% transcribing errors | ----- | Educational hospital | | | surgical and intensive care units | |
| Salmani et al., 2015 [[55](#_ENREF_55)] | N= 71 nurses | 47.9%  34(47.9%)of nurses/medication error | ----- | Educational hospital / non- Educational hospital | | | NICU | |
| Saremi et al., 2012 [[40](#_ENREF_40)] | N= 150 nurses | 53(34.8%) | ----- | Educational hospital | | | All ward | |
| Vazin et al., 2012 [[39](#_ENREF_39)] | N=38 patients | 442 errors per 5785 opportunities for errors (7.8%)/ | 9.8% administration errors, 6.8% prescribing errors, 3.3% transcription errors, 2.3% dispensing errors | teaching hospital | | | ICU ward | |
| Ahmadipour et al., 2015 [[53](#_ENREF_53)] | N= 293 medical residents & intern | 270/ 293 N =92.1% participants had committed medical error | Misdiagnosis (43.5- 54%), errors related to treatment (36.6-49%), history-taking and physical examination  (48.5-75%), follow-up care (22-31%),education of patients  (13-18.3%) | Educational hospital | | | ----- | |
| Bayazidi et al., 2012 [[21](#_ENREF_21)] | N= 733 nurses | mean (SD) of rate medication errors : 2.86(2.19) |  | Teaching hospitals | | |  | |
| [Saravi](https://www.ncbi.nlm.nih.gov/pubmed/?term=Saravi%20BM%5BAuthor%5D&cauthor=true&cauthor_uid=25870528)  et al., 2015 [[38](#_ENREF_38)] | N= 317966admissions | 182 cases (0.06%), medical error | Inappropriate and no care 68(37%)/ medication error 51(28%)/  Falling 21(11.5%)/  Equipment 5(2.7%)  Unknown 4(2.2%)  Others 33(18.2%) | non- teaching hospitals /  teaching hospitals | | | Internal medicine/ general surgery  /ICU  Radiology/pharmacy room  /emergency | |
| **Study /date** | **Sample** | **Prevalence of medical error** | **Type of medical error** | **Study location** | | | **study settings** | |
| Gavgani et al., 2013 [[17](#_ENREF_17)] | N= 140 medical records Patients | 16 (11%) patient medication errors | ----- | ----- | | | ----- | |
| Joolaee et al., 2011 [[22](#_ENREF_22)] | N= 286 nurses | average  1.3 | ----- | educational and no educational hospitals | | | ----- | |
| Khalili et al., 2011[[37](#_ENREF_37)] | N= 861 patients | 13.01% /112 medication errors among 861 patients | Dosing 44 (39.3%), choice 44 (39.3%), use 22 (19.7%), interaction problems 2 (1.7%). | referral teaching hospital | | | Infectious diseases ward | |
| Koohestani et al., 2009 [[36](#_ENREF_36)] | N= 240 nurses | 124 medication errors | 80.12% | educational hospitals | | | ----- | |
| Sadr et al., 2014 [[54](#_ENREF_54)] | N= 24 physicians | 24 cases of medical malpractice | ----- | educational and noneducational hospitals | | | ----- | |
| Akbari Sari et al., 2015 [[35](#_ENREF_35)] | N= 1162  hospital records | 128 (11.0%) | ----- | educational and noneducational hospitals | | | ----- | |
| Mohammadfam et al., 2015 [[33](#_ENREF_33)] | ---- | 53 error by SHERPA | Action=27(50.9%); checking=12(26.6%); communication=9(16%); retrieval=3(5.7%);  Selection=2 (3%) | ----- | | | ----- | |
| Kalantarzadeh et al., 2014 [[34](#_ENREF_34)] | N= 90 nursing students | mean of medication errors= 1.12 | Wrong dose=25 (41.7%)  Infusion rate=28 (46.7%) | educational hospitals | | | ----- | |
| **Study /date** | **Sample** | **Prevalence of medical error** | **Type of medical error** | **Study location** | | | **study settings** | |
| Valizadeh et al., 2008 [[32](#_ENREF_32)] | N= 898 medical charts |  | Wrong time= 14.8- 47.8% administration=74.1-77.5%,  dosage= 45.5% | educational hospitals | | |  | |
| Musarezaie et al., 2012 [[31](#_ENREF_31)] | N= 280 nurses | 13.6% | Wrong infusion speed=16(19%)  wrong dosage= 10(12%) | educational hospitals | | | Internal medicine/ general surgery | |
| Masror et al., 2012 [[30](#_ENREF_30)] | N= 200 nurses | 69.5% report from nurses | Medication errors (38.5%); skin and neuromuscular damage (36.7%), post-operative (16%) | educational and non-educational hospitals | | | ----- | |
| Mohsenzadeh et al., 2010 [[29](#_ENREF_29)] | N= 2250 Records | 6.2% (151/2250) | Administration errors = 46.3%  prescription errors =32.4%  transcription errors=10% | educational hospitals | | | ----- | |
| Ebrahimi et al., 2012 [[28](#_ENREF_28)] | N= 54 nurses | 184 | Wrong time= 65  administered error=21 | university | | | ----- | |
| Tabatabae et al., 2014 [[56](#_ENREF_56)] | N= 97 nurses | 572 | Medication without prescription=136(23.7%), lack of attention to medical complications=134(23.4%), Giving the medication at the wrong time=128(22.4%) | a nonpublic hospital | | | ----- | |
| Mohamad Nejad et al., 2010 [[27](#_ENREF_27)] | N= 78 Nurse | 37(17.9%) | Wrong dose=9  wrong medication =7  Wrong infusion speed=6 | educational hospitals | | | ----- | |
| Bozorgzad et al., 2014 [[26](#_ENREF_26)] | N=0400 medical chart | 50/400(12.5%) | 44% of the errors were occurred due to lack of timely medical care and 36 % of errors were related to report record of nursing | the Mortality Committee | | | ----- | |
| **Study /date** | **Sample** | **Prevalence of medical error** | **Type of medical error** | **Study location** | | | **study settings** | |
| Farzi et al., 2016 [[25](#_ENREF_25)] | N= 78 Nurse | (188/235)  80% of participants said that they have been experiencing medication errors over the past month | Wrong dose=88  wrong medication =29  Wrong time=20  administered error=27 | educational and noneducational hospitals | | | ICU | |
| Pourali et al., 2015 [[24](#_ENREF_24)] | N= 82  emergency physicians | 53 (63.8%) | Diagnostic errors= (48.2%) treatment errors= (33.3%) Prescription errors= 11.3%/ |  | | | emergency physicians | |
| Gahremani et al., 2016 [[23](#_ENREF_23)] | N= 74 nurse | 135/232 injection (58/19%) | Error in intravenous injection phase= 63%  Error in procurement procedure=36% | university hospital | | | ----- | |
| Yousefi et al., 2012 [[20](#_ENREF_20)] | N= 218 nurse | average medication errors= 31/6 | Wrong time | educational hospitals | | | medical and surgical wards | |
